# Diagnostic Potential of Plasma IgG N-glycans in Discriminating Thyroid Cancer from Benign Thyroid Nodules and Healthy Controls

**DOI:** 10.3389/fonc.2021.658223

**Published:** 2021-08-12

**Authors:** Zejian Zhang, Jianqiang Wu, Peng Liu, Lin Kang, Xiequn Xu

**Affiliations:** ^1^Department of Medical Research Center, Peking Union Medical College Hospital, Chinese Academy of Medical Sciences and Peking Union Medical College, Beijing, China; ^2^State Key Laboratory of Complex Severe and Rare Diseases, Peking Union Medical College Hospital, Chinese Academy of Medical Sciences and Peking Union Medical College, Beijing, China; ^3^Department of General Surgery, Peking Union Medical College Hospital, Chinese Academy of Medical Sciences and Peking Union Medical College, Beijing, China

**Keywords:** IgG N-glycosylation, bisecting type N-glycans, biomarker, mass spectrometry, thyroid nodules

## Abstract

**Background:**

Novel biomarkers are urgently needed to distinguish between benign and malignant thyroid nodules and detect thyroid cancer in the early stage. The associations between serum IgG N-glycosylation and thyroid cancer risk have been revealed. We aimed to explore the potential of IgG N-glycan traits as biomarkers in the differential diagnosis of thyroid cancer.

**Methods:**

Plasma IgG N-glycome analysis was applied to a discovery cohort followed by independent validation. IgG N-glycan profiles were obtained using a robust quantitative strategy based on matrix-assisted laser desorption/ionization time-of-flight mass spectrometry. IgG N-glycans were relatively quantified, and classification performance was evaluated based on directly detected and derived glycan traits.

**Results:**

Four directly detected glycans were significantly changed in thyroid cancer patients compared to that in non-cancer controls. Derived glycan traits and a classification glycol-panel were generated based on the directly detected glycan traits. In the discovery cohort, derived trait BN (bisecting type neutral N-glycans) and the glyco-panel showed potential in distinguishing between thyroid cancer and non-cancer controls with AUCs of 0.920 and 0.917, respectively. The diagnostic potential was further validated. Derived trait BN and the glycol-panel displayed “accurate” performance (AUC>0.8) in discriminating thyroid cancer from benign thyroid nodules and healthy controls in the validation cohort. Meanwhile, derived trait BN and the glycol-panel also showed diagnostic potential in detecting early-stage thyroid cancer.

**Conclusions:**

IgG N-glycome analysis revealed N-glycomic differences that allow classification of thyroid cancer from non-cancer controls. Our results suggested that derived trait BN and the classification glyco-panel rather than single N-glycans may serve as candidate biomarkers for further validation.

## Introduction

Thyroid cancer (TC) is the most common endocrine-related malignant tumor. Its incidence has increased significantly in recent years, with the global incidence at 6.7 percent, according to the data released by the Global Cancer Statistics 2018 (1). TC is mainly divided into four pathological types. Differentiated thyroid carcinoma (DTC), which originates from thyroid follicular epithelium, accounts for about 95% of TC. DTC includes papillary thyroid carcinoma (PTC) and follicular thyroid carcinoma (FTC), and more than 90% of malignant thyroid tumors are PTC (2, 3).

Epidemiological studies have shown that the palpation rate of thyroid nodules (TN) in women in the population is about 5% and that in men is about 1%. About 5% to 15% of the patients with TN will develop malignant lesions, which require timely thyroidectomy or other treatment such as central cervical lymph node resection, while the benign thyroid nodules (BTN) need standardized and regular follow-up (3–5). Therefore, the timely and accurate distinction between benign and malignant TN is of great clinical significance. What’s more, although DTC has a good overall prognosis, 6% to 13% of the lesions invade the important surrounding structures, and the proportion of distant metastasis is 5.9% to 23% at the time of diagnosis, leading to a significant decrease (26% ~ 70%) of the 10-year survival rate (5, 6). In general, most of these patients belong to stage III or IV TC patients at the time of diagnosis according to the TNM staging system of DTC (7). It has been reported that early diagnosis and timely treatment can significantly improve the survival rate of patients with tumors (8). Therefore, the early detection of TC is another key problem in clinical practice. However, no biomarkers are currently available for the differential diagnosis and early detection of TC. Ultrasound and ultrasound-guided percutaneous fine-needle aspiration (FNA) are important methods for preoperative evaluation of benign or malignant TN. However, these methods suffer from apparent shortcomings such as the need for professional operation, dependence on the experience of the clinicians, invasiveness, decreased accuracy for small nodules, cytological uncertainty present in 20% to 30% of TN, high cost, *etc.* (9–11). Summing up the above, it’s urgent to discover novel and non-invasive biomarkers that could complement ultrasound and FNA to improve the accuracy of discrimination between TC and non-cancer and are applicable for early detection of TC. It will be of great significance to avoid the overtreatment of benign thyroid patients and help malignant patients get a conclusive diagnosis and timely treatment in the early stage of cancer.

Glycosylation is one of the most common and important post-translational modifications of proteins. Glycans are involved in many key physiological and pathological processes such as carcinogenesis, cancer progression, and metastasis (12–14). Because glycans are involved in various cancer-related processes (cell differentiation, adhesion, invasion, metastasis, cell signaling, *etc.*), abnormal glycosylation is considered to be a hallmark of cancer (15). In addition to possible changes in the glycosylation of cancer-derived glycoproteins, there are also changes in the glycosylation of immunoglobulins (Igs) produced by B lymphocytes, suggesting that the changes in glycosylation are the result of a systemic response to tumorigenesis. Importantly, the changes can be detected in the blood. Glycans are potential biomarkers associated with systemic disorders of cancer patients (15). It has been increasingly reported that immunoglobulin G (IgG) glycosylation is associated with inflammation, immune dysfunction, and cancer (16–18). In our previous study, we found that dysregulation of IgG N-glycosylation was present in multiple types of cancer (19). In TC, the associations between N-glycosylation of human serum IgG fragment crystallizable (Fc) and TC risk have been revealed (20). What’s more, glycosylation of serum glycoproteins has been reported to change during TC development and progression (21). For example, two types of core-fucosylated glycans attached to the Fc region of serum IgG1 were changed during TC development (20, 21). In addition, sialylation of thyroid proteins is important to TC progression (21). Lectin histochemical staining of sialic acids in tissue specimens of human TC showed that transformation of thyroid follicular epithelial cells to PTC was associated with increased sialylation (21, 22). However, it’s still needed to be explored whether IgG glycans have the potential as novel biomarkers for TC early detection and differential diagnosis of benign or malignant TN.

In the present study, we performed a detailed analysis of the plasma IgG N-glycomic profiles in two cohorts (discovery and validation) consisting of TC, BTN, and healthy controls (HC) by employing a high-throughput quantitative strategy based on matrix-assisted laser desorption/ionization time-of-flight mass spectrometry (MALDI-TOF MS) (19, 23). We aimed to investigate and validate the potential of IgG N-glycans as biomarkers in the discrimination of TC patients from BTN and healthy individuals and detection of TC in early-stage.

## Materials and Methods

### Study Population and Sample Collection

One hundred and fifty-nine plasma samples of patients with benign and malignant TN and healthy participants were consecutively collected between June 2019 and February 2020 from Peking Union Medical College Hospital (Beijing, China). The collected samples were divided into a discovery cohort and a validation cohort. The discovery cohort was consisted of HC and patients with TC, while the validation cohort included three groups of participants: HC, patients with BTN, and patients with TC. The subgroups were age- and sex-matched as far as possible in each cohort. Healthy cases were defined by medical doctors according to eligibility criteria. HC should have no history of systematic diseases and have normal thyroid ultrasound, thyroid function, and biochemical parameters. Patients with benign and malignant TN were diagnosed on the basis of ultrasound and FNA and were confirmed by surgical histopathology. All patients with TC were clinically classified as PTC. We obtained approval from the regional ethics committee of Peking Union Medical College Hospital and informed written consents from all participants were acquired. More detailed information on the cohorts was summarized in **Table 1**.

**Table 1:** Clinicopathological characteristics of all participants by subgroup.

Study samples for the differential diagnosis of benign and malignant TN							
	Discovery cohort (n = 50)			Validation cohort (n = 109)			
	TC (n = 25)	HC (n = 25)	*p*	TC (n = 47)	HC (n = 44)	BTN (n = 18)	*p*
**Age, mean (range)**	38.48 (26-57)	37.08 (27-52)	0.776	38.47 (22-63)	37.43 (23-51)	40.11 (18-63)	0.598
**Gender, (male)%**	25%	25%	1.000	43%	52%	40%	0.525
**Pathological types**	PTC	—	—	PTC	—	—	—
**Study samples for detection of early-stage TC**							
	**Samples (discovery + validation cohort)**			**Age- and sex- matched subgroups (after exclusion of some male non-cancer controls and advanced TC)**			
	Early-stage TC (n = 30)	Advanced-stage TC (n = 42)	Non-cancer control (n = 87)	Early-stage TC (n = 30)	Advanced-stage TC (n = 28)	Non-cancer control (n = 65)	*P*
**Age, mean (range)**	41.1 (25-63)	36.6 (22-57)	37.9 (18-63)	41.1 (25-63)	38.5 (22-57)	38.6 (23-63)	0.447
**Gender, (male)%**	17%	47%	40%	17%	21%	20%	0.951

TC, thyroid cancer; HC, healthy controls; BTN, benign thyroid nodules.

### IgG Purification From Human Plasma

Protein A Spin Plate for IgG Screening (Thermo Fisher Scientific, Rockford) was utilized in this study to isolate and purify IgG from blood samples. IgG was purified from each plasma sample as previously described (19). Briefly, 70 µL of blood plasma was first diluted using Protein A IgG Binding Buffer (Thermo Fisher Scientific, 0.5L). The diluted samples were applied to the Spin plate wells and washed five times by Binding Buffer to thoroughly wash away all unbound non-IgG protein components. Last, the bound IgG was eluted by Elution Buffer (Thermo Fisher Scientific, Rockford, 0.5L) three times into three separate collection plates. In order to determine which fractions contained IgG, the absorbance was measured for each fraction at 280 nm by bicinchoninic acid (BCA) test (Thermo Fisher Scientific, 1L). The purity of eluted IgG was validated by sodium dodecyl sulfate-polyacrylamide gel electrophoresis (SDS-PAGE) (**Figure S1**). The fractions containing IgG were stored at −20°C until the release of N-glycans.

### IgG N-Glycans Release and Enrichment

Fifty µL of denatured IgG-containing fractions of Protein A column elution were used for N-glycans release. According to the protocols of our previous paper (19, 23), IgG N-glycans were released by mixing the IgG elution with PNGase F (New England Biolabs, Inc., USA) followed by 12 h incubation at 37°C. IgG N-glycans were enriched and purified from the enzyme solution by using a PGC-containing 96-well plate as described previously (19, 23). In brief, the PGC-containing 96-well plate was first conditioned with 0.1% trifluoroacetic acid (TFA) in 80% acetonitrile (ACN), followed by 0.1% TFA in H_2_O. The solution of the enzyme-released glycans was subsequently loaded to the PGC-containing 96-well plate three times to allow complete N-glycans binding. Then the plate was washed with H_2_O to remove the salts and other contaminants. The N-glycans derived from IgG were finally eluted into the collection plate using 0.05% TFA in 25% ACN.

### MALDI-TOF MS Analysis

Before sample detection, a calibration standard of peptides (Calibration Standard II, Bruker Daltonics, Bremen, Germany) was used for external calibration of the instrument. The IgG N-glycans were analyzed by MALDI-TOF MS as previously described with minor modification (19, 23, 24). Briefly, one μL of purified glycans was spotted onto an MTP BigAnchor 384BC MALDI target plate (Bruker Daltonics). Then one μL of matrix consisting of 5 mg/mL super-DHB in 50% ACN was mixed with the sample on the plate and allowed to dry by air, followed by recrystallization with pure ethanol. All mass spectrometry analyses were performed on a rapifleX MALDI-TOF mass spectrometer with a Smartbeam 3D laser in reflection positive (RP) mode, controlled by flexControl 4.0 (Bruker Daltonics). Sample measurements were performed with a mass range from m/z 1000 to m/z 3500. For each spectrum, 3000 laser shots were accumulated using a complete sample random walk with 200 shots per raster spot at a laser frequency of 5000 Hz.

### Data Processing and Statistical Analysis

The pre-processing of MALDI-TOF MS data was performed according to our previous work (19, 23, 24). Briefly, MALDI-TOF MS spectra of the two cohorts were smoothed, baseline corrected, and transformed to.XY format by using flexAnalysis software (Bruker Daltonics). Using the in-house developed software MassyTools (version 0.1.8.1.2) (25), transformed.XY profiles of the cohorts were re-calibrated. Next, we used the Glyco-Peakfinder tool of GlycoWorkbench (26, 27) to generate putative glycan compositions, and the putative glycan compositions were validated by previous literature (23). Of note, if multiple cation adduct signals such as [M + Na]^+^ and [M + K]^+^ were present simultaneously, all these related adducts should be included in the quantification (23). Detailed information on glycan identification was provided in the **Supplemental Information**. The intensities for the putative N-glycan structures were finally extracted as background-corrected area for each spectrum of the samples using MassyTools. Derived glycan traits including Gal-ratio (representing the level of IgG galactosylation), fucosylation (F), agalactosylation (agal), and bisecting type neutral N-glycans (BN) were calculated based on the same structural features (**Supplemental Table S1**). A glyco-panel was constructed by combining altered detected N-glycans between cases and controls through logistic regression in SPSS (version 19.0). In this process, the group was set as the dependent variable, and the altered glycan traits were set as concomitant variables. Prediction probability generated during the logistical regression process was defined as glyco-panel. The following statistical analyses were performed with GraphPad Prism 7 and SPSS. Data quality was assessed and controlled by the inclusion of six technical replicates of a standard plasma sample and calculating the average value, standard deviation (SD), and the relative SD (CV) for all detected glycans. The diagnostic potential of the IgG N-glycans detected in the present study was assessed by performing statistical tests and receiver-operator-characteristics (ROC) analysis. Differences between two biological groups were assessed using the Mann-Whitney U test (non-normally distributed data), and the significance threshold of p-values is 0.004 (= p-value of 0.05 after multiple testing correction for 12 glycan traits). Differences between the three groups were evaluated by one-way analysis of variance (ANOVA) with Bonferroni correction for multiple comparisons using GraphPad Prism 7. Glycan traits resulting in statistically significant p-values were further evaluated by ROC tests to assess the specificity, sensitivity, and accuracy of the candidate IgG N-glycans using GraphPad Prism 7. The significance of the generated values of area-under-the-curve (AUC) was assigned. If the AUC values were greater than 0.9, the tests were considered “highly accurate,” while values between 0.8 and 0.9 were deemed “accurate.” When the AUC values were between 0.7 and 0.8, the tests were concluded to be “moderately accurate”. An “uninformative” test resulted in an AUC value that was between 0.5 and 0.7 (23, 28).

## Results

### Plasma IgG N-Glycan Profiles by MALDI-TOF MS

**Figure 1:**
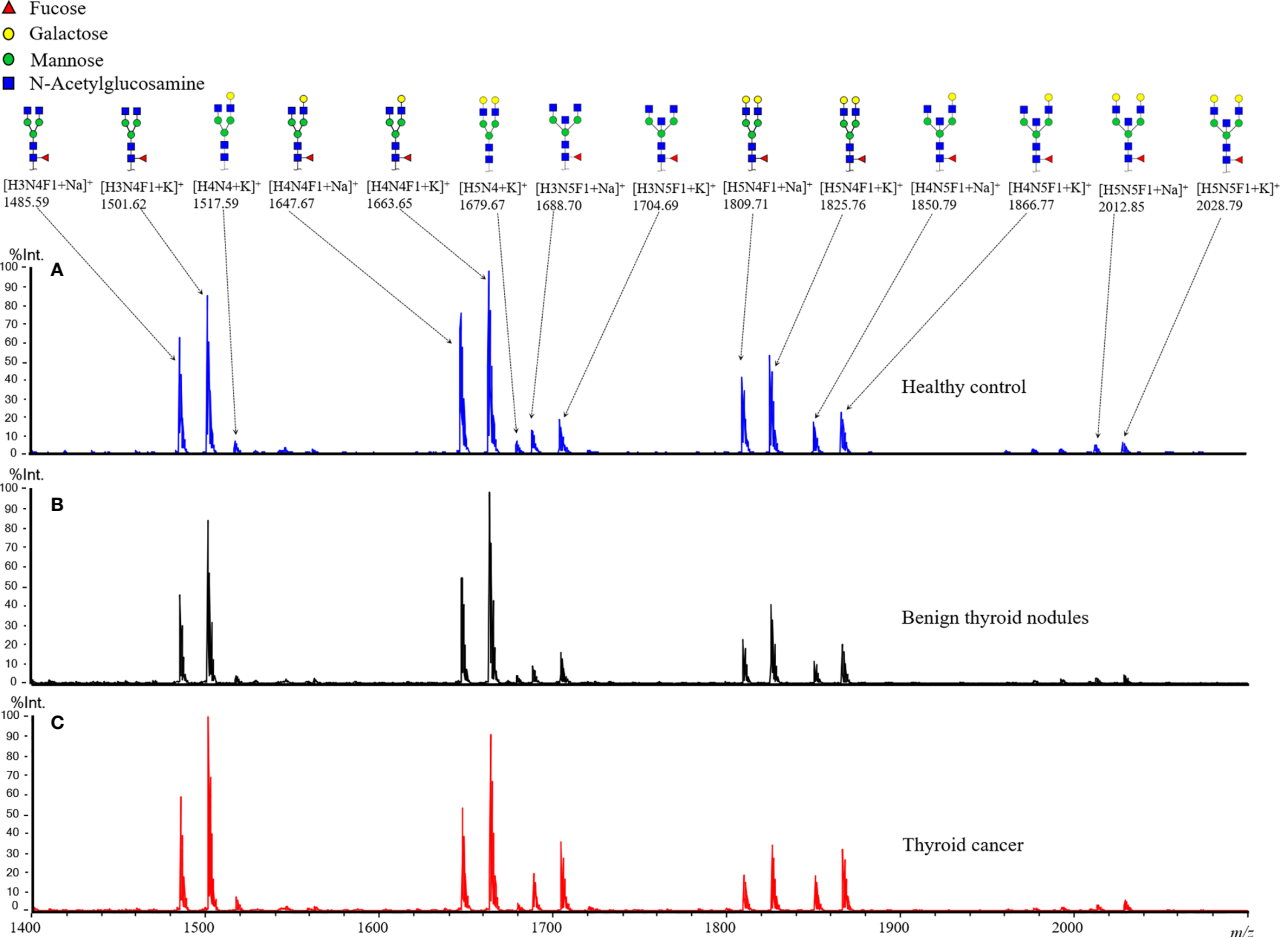
Representative MALDI-TOF MS spectra of plasma IgG N-glycan profiles for **(A)** healthy control, patients **(B)** with benign thyroid nodules, and **(C)** with thyroid cancer. Spectra were recorded in positive ion reflectron mode on a rapifleX MALDI-TOF mass spectrometer with a Smartbeam 3D laser. Major glycan peaks were annotated and assigned to compositions and structural schemes. H, hexose; N, N-acetylhexosamine; F, deoxyhexose (fucose).

In the present study, the plasma IgG N-glycans were profiled by MALDI-TOF MS. The typical annotated MALDI-TOF MS spectra of IgG N-glycomic profiles from plasma samples of TC, BTN, and HC were shown in **Figure 1**. In our obtained data, eight N-glycan compositions were directly detected and quantified (**Supplemental Table S1**). In addition, four derived glycan traits, including Gal-ratio, F, agal, and BN, were calculated based on the same structural features (**Supplemental Table S1**). For the six technical replicates of the quality-control plasma standard, the CVs of all glycan compositions were below 10%, which means the quality of the acquired data was reliable (**Supplemental Table S2**).

### IgG N-Glycomic Changes in TC

**Figure 2:**
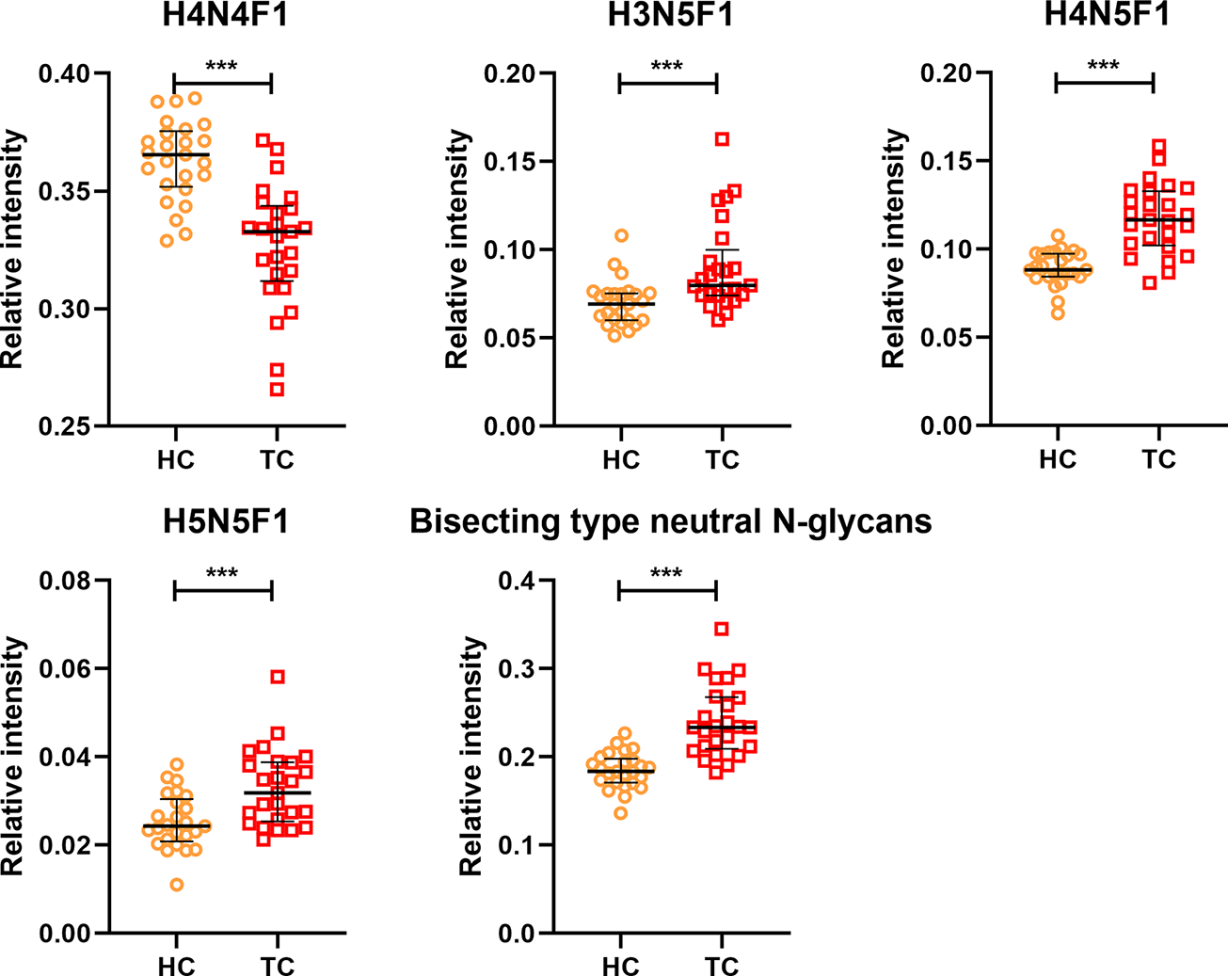
The scatter plots of main directly detected and derived glycan traits in thyroid cancer (TC) and healthy controls (HC) for the discovery cohort. The whiskers represent “median with IQR”. ***p-value < 0.001 (after Bonferroni correction).

**Figure 3:**
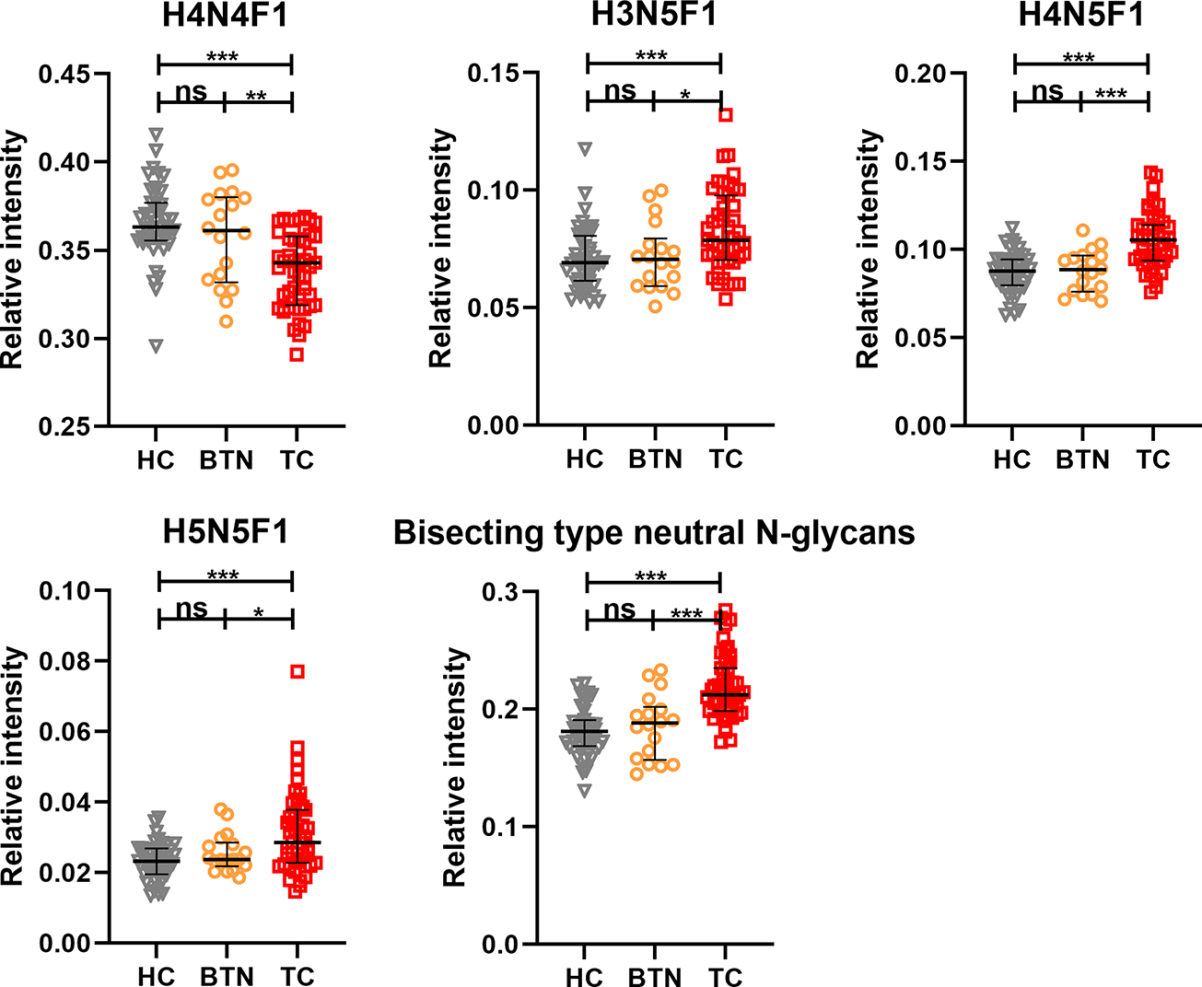
The scatter plots of main replicated directly detected and derived glycan traits in thyroid cancer (TC), benign thyroid nodules (BTN) and healthy controls (HC) for the validation cohort. The whiskers represent “median with IQR”. ***p-value < 0.001, **p-value < 0.01, *p-value < 0.05, ns, not significant (after Bonferroni correction).

In order to profile the differentially expressed IgG N-glycans between TC and non-cancer, eight directly detected glycan traits and four derived glycan traits were compared between TC patients (n=25) and HC individuals (n=25) in the discovery cohort. Significant differences were observed in four directly detected glycan traits (**Figure 2** and **Supplemental Table S3**). H4N4F1 (G1) was significantly decreased in TC patients compared with HC (P<0.0001). Relatively, higher levels were found in TC patients than that in HC for H3N5F1, H4N5F1, and H5N5F1 (P=0.0004, P<0.0001, P=0.001, **Figure 2** and **Supplemental Table S3**). The three elevated directly detected glycan traits in TC all belong to the bisecting type. Consistently, derived glycan trait BN was increased in TC than that in HC in the discovery cohort (P<0.0001, **Figure 2** and **Supplemental Table S3**). However, for other directly detected and derived glycan traits, including H3N4F1, H4N4, H5N4, H5N4F1, F, agal, and Gal-ratio, no differences were found between TC and HC (**Supplemental Table S3** and **Supplemental Figure S2**). In order to validate the N-glycomic changes found in the discovery cohort and to explore whether there also exists differentially expressed IgG N-glycans between TC and BTN, IgG N-glycans were profiled and compared in 47 patients with TC, 18 patients with BTN and 44 HC individuals in the validation cohort. Consistently, we found that there were significant differences between TC and BTN for H4N4F1, H3N5F1, H4N5F1, H5N5F1, and derived glycan trait BN, similar to the results when comparing TC to HC (**Figure 3** and **Supplemental Table S3**, **Supplemental Figure S3**). However, we did not find differences in these N-glycan traits between BTN and HC (**Figure 3** and **Supplemental Table S3**, **Supplemental Figure S3**).

As far as we know, this is the first time that IgG N-glycomic profiles have been investigated in BTN samples. Our results suggested that there existed differentially expressed IgG N-glycans between TC and non-cancer controls (HC+BTN), and these changed glycan traits (directly detected + derived) might be used to distinguish TC from BTN and HC, which will be evaluated in the next part of this study.

### Performance of IgG N-Glycans in Discriminating TC From BTN and HC

**Figure 4:**
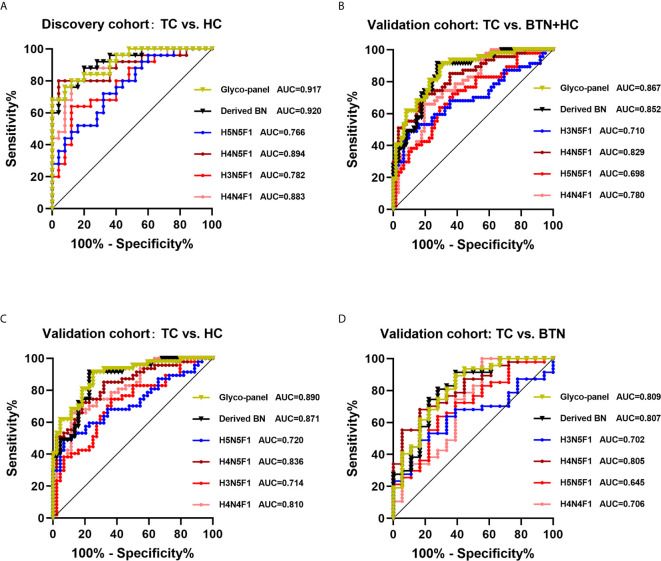
ROC curve analysis for differential expressed IgG glycan traits and a glyco-panel built based on altered single glycan traits **(A)** in the discovery cohort and **(B–D)** in the validation cohort. In the discovery cohort, we performed analysis between TC and HC. In the validation cohort, we performed analysis among three subgroups (TC *vs*. HC+BTN, TC *vs*. HC, TC *vs*. BTN). TC, thyroid cancer; HC, healthy controls; BTN, benign thyroid nodules.

The performance of differentially expressed IgG N-glycan traits mentioned above in discriminating TC from BTN and HC was further evaluated by the ROC curve in the discovery cohort, and the results were validated in an independent validation cohort. For the directly detected glycan traits, the AUCs of H4N4F1, H3N5F1, H4N5F1 and H5N5F1 were 0.883 (95%CI: 0.790–0.976), 0.782 (95%CI: 0.656–0.909), 0.894 (95%CI: 0.802–0.987), and 0.766 (95%CI: 0.638–0.895) when detecting TC in the discovery cohort (**Figure 4A**). Although these differentially expressed directly measured single N-glycans had acceptable performance, a glyco-panel constructed from these altered directly detected glycan traits using a logical algorithm might have a better performance for differential diagnosis, considering these N-glycans showed strong associations with TC. Therefore, we built a glyco-panel consisting of H4N4F1, H3N5F1, H4N5F1, and H5N5F1 using logistic regression in the discovery cohort. The performance of the glyco-panel was improved with an AUC of 0.917 (95%CI: 0.844–0.990), a sensitivity of 80.00%, and a specificity of 88.00% compared with the single N-glycan traits (**Table 2** and **Figure 4A**). Of note, the performance of the glyco-panel was similar to that of derived glycan trait BN with an AUC of 0.920 (95%CI: 0.848–0.992), a sensitivity of 88.00%, and a specificity of 80.00% (**Table 2** and **Figure 4A**). These results were verified in the validation cohort (**Table 2** and **Figures 4B–D**). The glycol-panel and derived trait BN displayed “accurate” performance (AUC>0.8) in discriminating TC from BTN and HC (TC *vs*. HC, TC *vs*. BTN, and TC *vs*. BTN+HC) in the validation cohort (**Table 2** and **Figures 4B–D**), indicating that the glyco-panel and derived glycan trait BN of IgG may have potential as biomarkers in discriminating TC from BTN and HC.

**Table 2:** The accuracy, sensitivity, and specificity of the glyco-panel and derived glycan trait BN (bisecting type neutral N-glycans) for diagnosis of TC in discovery and validation cohort.

	Discovery cohort (n = 50)		Validation cohort (n = 109)						Detection of early-stage TC	
			TC *vs*. BTN+HC		TC *vs*. BTN		TC *vs*. HC			
	Glyco-panel	Derived trait BN	Glyco-panel	Derived trait BN	Glyco-panel	Derived trait BN	Glyco-panel	Derived trait BN	Glyco-panel	Derived trait BN
**Accuracy (AUC)**	0.917 (95%CI: 0.844–0.990)	0.920 (95%CI: 0.848–0.992)	0.867 (95%CI: 0.800–0.933)	0.852 (95%CI: 0.783–0.922)	0.809 (95%CI: 0.685–0.932)	0.807 (95%CI: 0.682–0.933)	0.890 (95%CI: 0.825–0.955)	0.871 (95%CI: 0.799–0.943)	0.907 (95%CI: 0.833–0.981)	0.896 (95%CI: 0.824–0.968)
**Sensitivity**	80.00%	88.00%	89.36%	91.49%	89.36%	80.85%	91.49%	91.49%	80.00%	80.00%
**Specificity**	88.00%	80.00%	70.97%	72.58%	61.11%	72.22%	75.00%	77.27%	94.25%	87.36%

For the discovery cohort, the analysis was performed between TC and HC. For the validation cohort, the analysis was performed between TC and non-cancer (TC vs. HC, TC vs. BTN, and TC vs. BTN+HC). TC, thyroid cancer; HC, healthy controls; BTN, benign thyroid nodules.

### Performance of IgG N-Glycans in Detection TC at Early Stage

It has been reported that early diagnosis and timely treatment can significantly improve the survival rate of patients with malignant tumors. Thus, the differential levels of IgG derived glycan trait BN and the glyco-panel between the early-stage of TC and the non-cancer group were assessed. We also evaluated whether the glyco-panel and derived glycan trait BN have the potential as biomarkers to detect early-stage TC by ROC tests. Of note, in the present study, TC without lymphatic metastasis was taken as early-stage cancer. The non-cancer controls consisted of HC and BTN from the discovery and validation cohort. Early/advanced TC involves patients from both the discovery and validation cohort (**Table 1**).

**Figure 5:**
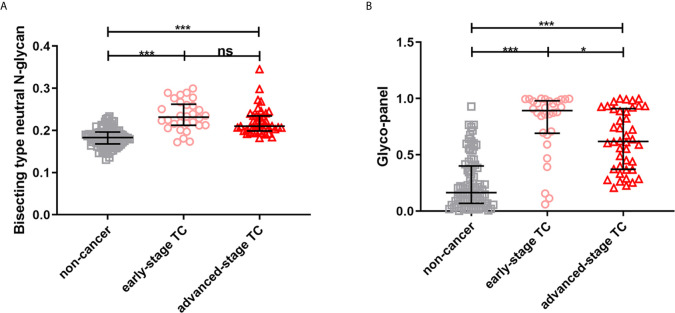
The scatter plots of **(A)** derived glycan trait BN and **(B)** the glyco-panel in early- and advanced-stage thyroid cancer (TC) and non-cancer controls. The whiskers represent “median with IQR”. ***p-value < 0.001, *p-value < 0.05, ns, not significant (after Bonferroni correction).

**Figure 6:**
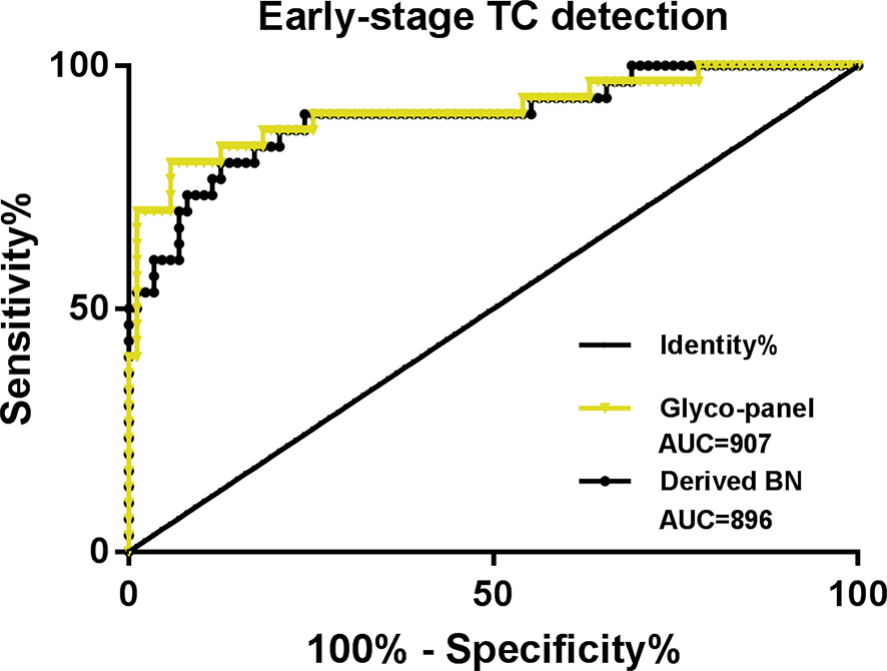
ROC curve analysis for derived glycan trait BN and the established glyco-panel in detecting early-stage thyroid cancer (TC).

The results showed that the glyco-panel and derived glycan trait BN were significantly different between early-stage TC and non-cancer (**Figure 5**). Further, for the ROC tests, the AUCs of glyco-panel and derived glycan trait BN were 0.907 (95%CI: 0.833–0.981; sensitivity: 80.00%; specificity: 94.25%) and 0.896 (95%CI: 0.824–0.968, sensitivity: 80.00%; specificity: 87.36%) in discriminating patients with early-stage cancer from non-cancer controls (**Table 2** and **Figure 6**). What is noteworthy is that the subgroups of early-stage TC and non-cancer control used above in ROC analysis were not matched for gender. In order to reduce the influence of gender on the results, we randomly excluded some samples from the male individuals in the non-cancer control group. Then we performed ROC analysis in the age- and sex- matched subgroups of early-stage TC (n=30) and non-cancer controls (n=65) (**Supplemental Table S4**). Our results showed that derived trait BN and the glycol-panel still displayed considerable performance (**Supplemental Figure S4**). Thus, the glyco-panel and derived glycan trait BN of IgG may have the potential to be used as biomarkers for detection of early-stage TC assisting the existed ultrasound and FNA.

## Discussion

TC is the most common endocrine tumor and accurate early diagnosis is helpful for subsequent intervention. Currently, no biomarkers are available for the diagnosis of TC. Differential diagnostic methods used in clinical practice to distinguish between benign and malignant TN suffer from obvious shortcomings. Thereby, it is urgently needed to explore novel biomarkers to diagnose TC more conclusively.

The analysis of glycomic profiles of relevant glycoproteins in serum/plasma provides a new approach for the discovery of non-invasive tumor biomarkers. So far, aberrant glycosylation in TC has been reported in several studies. Employing MALDI-TOF(/TOF)-MS, researchers found six N-glycan compositions, including four sialylated N-glycans and two high-mannose type N-glycans, were significantly different (p < 0.001) between formalin-fixed paraffin-embedded (FFPE) tissues of patients diagnosed with PTC and controls (29). What’s more, it was suggested that glycosylation is important in controlling TC development and progression (21). However, there were limited studies on IgG glycosylation of TC, although it has shown potential in biomarker screening (20, 21).

IgG is one of the most abundant glycoproteins in circulation, with a serum concentration ranging from 7 to 18 mg/mL in healthy adults (16). Dysregulation of IgG glycosylation has been revealed in various cancers including TC (19, 20, 30–34). In our previous study, we found the distribution of IgG galactosylation referred to IgG Gal-ratio had a common feature and was increased in multiple types of cancer (not including TC) compared to non-cancer controls. IgG Gal-ratio showed great potential for cancer screening (19). Chen et al. reported human serum IgG Fc glycosylation was associated with TC risk (20). However, IgG N-glycomic profiles have never been investigated in BTN. Of note, benign or malignant TN are sometimes difficult to be distinguished using the present methods. Misdiagnosis of benign or malignant TN can bring psychological burden and unnecessary surgery for the patients with benign TN and delay the timely treatment for malignant TN.

In this study, we profiled the IgG N-glycosylation in TC, BTN, and HC simultaneously for the first time. We found differentially expressed IgG N-glycans between BTN and TC by statistical tests. In addition, we did not find differences in glycan traits between BTN and HC. ROC analysis demonstrated that derived glycan traits BN and a glycol-panel have potential in discriminating TC from BTN and HC (AUC>0.8). However, the potential of IgG glycans as differential diagnostic biomarkers for discriminating TC from BTN still needs to be validated in larger cohorts.

Furthermore, there were some differences in AUC, sensitivity, and specificity between the discovery and validation cohort. In the present study, the discovery cohort consisted of two subgroups (HC and TC), while the validation cohort consisted of HC, BTN, and TC. We combined BTN and HC as the non-cancer group for the ROC analysis (**Figure 4B**) in the validation cohort. Though there were no statistically significant differences between BTN and TC in glycan traits (maybe due to the sample size of BTN), the inclusion of benign disease in the control group showed some influence on the AUC, sensitivity, and specificity, and BTN may be an intermediate state between HC and TC.

It’s worth noting that abnormal glycosylation, including derived trait BN and the glycol-panel, was already present in early-stage TC, which indicates the potential of these glycan traits to discriminate early-stage TC from non-cancer controls. However, derived trait BN displayed no significant differences between early- and advanced- stage TC, suggesting that BN showed no utility for monitoring the progression of TC. It is interesting to note that glycol-panel decreased from early- to advanced- stage TC. The in-depth mechanisms (e.g., the changes in glycosyltransferases during the progression of TC) behind the changes still need investigation, preferably in a prospective manner. In addition, though the glycol-panel and derived glycan trait BN showed potential in detecting early-stage TC and the accuracy is 0.907 and 0.896, respectively, the sensitivity is relatively low (80.00%), which means false negatives can occur. The performance of these glycan traits may not be sufficient in clinical practice, suggesting that glycan traits in conjunction with other diagnostic modalities (such as ultrasound and FNA) rather than used separately may improve diagnostic accuracy and sensitivity and meet the clinical needs.

As for the mechanisms, IgG is known to play essential roles in humoral immunity and is involved in humoral immune processes such as complement-dependent cytotoxicity (CDC) and antibody-dependent cell-mediated cytotoxicity (ADCC) (16, 35). The structure and activity of IgG are affected by its fragment crystallizable region (Fc) glycosylation. Furthermore, IgG alternative glycosylation modulates the functions of IgG effectors and is involved in the development and progression of disease (16, 36). Increased occurrence of bisecting GlcNAc and a lack of core fucose and galactose increase the ability of IgG to induce ADCC (16, 35). In the present study, total bisecting GlcNAc of neutral N-glycans (BN) were increased in patients with TC than non-cancer controls, suggesting ADCC activity might be increased in TC. Interestingly, we did not find a significant difference between case and control for IgG Gal-ratio (level of galactosylation) (**Supplemental Table S2**), which showed increased levels in multiple types of cancer in our previous study (19). This may indicate that the mechanisms of dysregulated IgG glycosylation in TC patients are to some extent unique under the influence of endocrine and that IgG glycosylation in patients with TC may interact with hormones. Consistently, previous reports also provide evidence linking hormones with IgG glycosylation (29, 37, 38). Chen et al. reported associations between sex hormones (including estradiol and progesterone) and IgG glycans in TC (20). They also found that digalactosylated glycan features appeared to have a stronger correlation with sex hormones than the monogalactosylated ones, which may explain the effects on Gal-ratio (20). Additionally, serum estradiol level in patients with TC was found higher than that in controls (20). On the other hand, they revealed that the observed association of IgG glycosylation with sex hormones might explain some of the differences in morbidity of TC between males and females. Consistently, Ercan et al. also found that estrogens regulate IgG glycosylation in males and females (37). In addition, progesterone influences the expression of oligosaccharyltransferase with marked consequences on IgG N-glycosylation (38). Due to the complexity of the sex hormone control system and the regulation of IgG glycosylation, future efforts should be infused to elucidate how the hormonal effects on IgG glycosylation are mediated in TC. The underlying mechanisms and causes of the aberrant IgG glycosylation in TC remain thus far unknown. It has been reported that the changes of IgG glycosylation may be related to the inflammatory state in cancer patients (16, 39, 40). Particularly, hormones may also be involved in the regulation of glycosylation in TC (20, 29). Thus, we hypothesize that the aberrant IgG glycosylation features in TC are the result of a combination of immune disorder and hormones, which needs further exploration in the future.

In this study, we investigated IgG N-glycans in TC, HC, and BTN using a high-throughput method based on MALDI-TOF MS. MALDI-TOF MS has several advantages, such as high sensitivity, high resolution, and high speed. The workflow we used in the present study is robust and has also been widely used in several other studies for IgG glycosylation profiling (19, 23, 41–43). Moreover, we included a standard sample randomly distributed in two 96-well sample plates for quality control. The CVs of all glycans of this standard sample were all below 10%, indicating the quality of the data was sufficiently reliable. In addition, the workflow is high-throughput and convenient for further clinical application. However, our study has several limitations. First, in the present study, we merely focused on neutral glycans, while IgG sialylated glycans and IgG subclass-specific glycans still need to be investigated in TC and BTN by other approaches. Second, patients with BTN are the disease control in the present study. In order to ensure that the nodules are benign, all the BTN should be confirmed by surgical pathology in this study. So it is not easy for us to obtain equal plasma samples from BTN as from TC. However, for a fair comparison, subgroup sample sizes should be (more) equal. The sample size of the validation cohort is not large enough, which limited the power of our analyses. This is one of the limitations of the present study. Third, as the sample size of early-stage TC in each cohort was not large enough, we combined early-stage TC of the discovery and validation cohort for statistical test and ROC analysis. The results for early-stage TC detection still need validation in a larger independent cohort. Fourth, it has been reported that IgG glycosylation is associated with age and sex. We matched age and gender as far as possible between groups in each cohort during our study design. Nevertheless, due to the sample size of BTN is not large enough, we were unable to conduct stratified analyses according to age and sex. Therefore, before the potential candidate biomarkers for TC we found in this study could be applied clinically, stratified analyses of subgroups according to age and sex and prospective investigation in larger cohorts are required in future studies.

In conclusion, we profiled IgG N-glycosylation in TC, HC, and BTN simultaneously applying a rapid, robust and high-throughput workflow through MALDI-TOF MS and found differentially expressed IgG (directly detected + derived) N-glycan traits between TC and non-cancer control. To our knowledge, this is the first attempt that IgG N-glycomic profiles have been quantitatively evaluated in BTN. Further, we established and validated candidate IgG glycan biomarkers (a glycol-panel and derived glycan trait BN) to differentially diagnose benign or malignant TN as well as to detect early-stage TC. Further studies into the roles of protein glycosylation in the pathology of TC and prospective investigation and validation in larger cohorts are still needed.

## Data Availability Statement

The original contributions presented in the study are included in the article/**Supplementary Material**. Further inquiries can be directed to the corresponding author.

## Ethics Statement

The studies involving human participants were reviewed and approved by the regional ethics committee of Peking Union Medical College Hospital. The patients/participants provided their written informed consent to participate in this study.

## Author Contributions

XX and ZZ conceived and initiated this study. ZZ performed the experiments and data analysis and interpreted the results with support of XX, JW, KL, and PL. ZZ, JW, LK, and PL collected samples and clinical parameters. ZZ prepared the figures and tables, and wrote the original draft with support from XX. All authors contributed to the article and approved the submitted version.

## Funding

The work was supported by the National Natural Science Foundation of China (32071436, 31901041).

## Conflict of Interest

The authors declare that the research was conducted in the absence of any commercial or financial relationships that could be construed as a potential conflict of interest.

## Publisher’s Note

All claims expressed in this article are solely those of the authors and do not necessarily represent those of their affiliated organizations, or those of the publisher, the editors and the reviewers. Any product that may be evaluated in this article, or claim that may be made by its manufacturer, is not guaranteed or endorsed by the publisher.

## Abbreviations

ACN, acetonitrile; ADCC, antibody-dependent cell-mediated cytotoxicity; agal, agalactosylation; ANOVA, one-way analysis of variance; AUC, area-under-the-curve; BCA, bicinchoninic acid; BN, bisecting type neutral N-glycans; BTN, benign thyroid nodules; CDC, complement-dependent cytotoxicity; DTC, differentiated thyroid carcinoma; F, fucosylation; Fc, fragment crystallizable; FFPE, formalin-fixed paraffin-embedded; FNA, fine-needle aspiration; FTC, follicular thyroid carcinoma; HC, healthy controls; IgG, immunoglobulin G; Igs, immunoglobulins; MALDI-TOF MS, matrix-assisted laser desorption/ionization time-of-flight mass spectrometry; PTC, papillary thyroid carcinoma; ROC, receiver-operator-characteristics; RP, reflection positive; SD, standard deviation; SDS-PAGE, sodium dodecyl sulfate-polyacrylamide gel electrophoresis; TC, thyroid cancer; TFA, trifluoroacetic acid; TN, thyroid nodules.
