# Supplementary material for: Diagnostic Potential of Plasma IgG N-glycans in Discriminating Thyroid Cancer from Benign Thyroid Nodules and Healthy Controls
Source: Front Oncol. 2021 Aug 12;11:658223. doi: 10.3389/fonc.2021.658223 (PMC8406750; doi:10.3389/fonc.2021.658223)
Supplement: Supplementary file 1 [file DataSheet_1.pdf]

Supplemental Information

Diagnostic Potential of Plasma IgG N-glycans in Discriminating Thyroid Cancer from Benign Thyroid Nodules and Healthy Controls

Zejian Zhang[1,2], Jianqiang Wu[1,2], Peng Liu[1,2], Lin Kang[1,2], Xiequn Xu[2,3*]

[1]Department of Medical Research Center, Peking Union Medical College Hospital, Chinese Academy of Medical Sciences and Peking Union Medical College, Beijing, China
[2]State Key Laboratory of Complex Severe and Rare Diseases, Peking Union Medical College Hospital, Chinese Academy of Medical Sciences and Peking Union Medical College, Beijing, China
[3]Department of General Surgery, Peking Union Medical College Hospital, Chinese Academy of Medical Sciences and Peking Union Medical College, Beijing, China

* Correspondence:
Xiequn Xu
xxq75@163.com

Detailed Protocols

Study Population and Sample Collection

One hundred and fifty-nine plasma samples from patients with benign and malignant thyroid nodules (TN) and healthy control (HC) individuals were consecutively collected between June 2019 and February 2020 from Peking Union Medical College Hospital (Beijing, China). The samples collected were divided into discovery and validation cohorts. The discovery cohort was consisted of HC and patients with thyroid cancer (TC), while the validation cohort included three groups of participants: HC, patients with benign thyroid nodules (BTN), and patients with TC. The groups were age- and sex-matched as far as possible in the respective cohort. Healthy cases were defined by medical doctors according to eligibility criteria, and they should have no history of systematic diseases, have normal thyroid ultrasound, normal thyroid function, and biochemical parameters. Patients with benign and malignant TN were diagnosed on the basis of ultrasound and fine-needle aspiration (FNA) and were confirmed by surgical histopathology. All patients with TC were clinically classified as papillary thyroid carcinoma (PTC). We obtained approval from the regional ethics committee of Peking Union Medical College Hospital, and informed written consents from all participants were acquired.

IgG Purification from Human Plasma

Protein A Spin Plate for IgG Screening (Thermo Fisher Scientific, Rockford) was utilized in this study to isolate and purify IgG from blood samples. IgG was purified from each plasma sample as previously described (1, 2). Briefly, to equilibrate the Protein A Spin Plate, Binding Buffer was added to each well. After that, the Spin plate was placed assembly onto a centrifuge with a 96-well plate carrier and centrifuged for 1 minute at 1,000 × g. Then, 70 μL of blood plasma was diluted using 70 μL of Protein A IgG Binding Buffer (Thermo Fisher Scientific, 0.5L), and the diluted samples were applied to the Spin plate wells followed by 30 mins incubation with moderate agitation. Next, the Spin plate was centrifuged assembly at 1,000 × g for 1 minute. Subsequently, the Spin plate was washed five times by Binding Buffer to wash away all unbound non-IgG protein components thoroughly. Last, the bound IgG was eluted by Elution Buffer (Thermo Fisher Scientific, Rockford, 0.5L) three times into three separate collection plates. In order to determine which fractions contained IgG, the absorbance was measured for each fraction at 280 nm by bicinchoninic acid (BCA) test (Thermo Fisher Scientific, 1L). The fractions containing IgG were stored at −20 °C until the release of N-glycans.

IgG N-glycans Release and Enrichment

IgG N-glycans were released according to the protocols of our previous papers (1, 2). Briefly, a 96-well PVDF membrane plate was prewashed with 70% ethanol/H_2O and water. Fifty μL of denatured IgG-containing fractions of Protein A column elution were used for N-glycans release and added into each well of the prewashed 96-well PVDF membrane plate. Next, the release mixture

containing 1 U PNGase F (New England Biolabs, Inc., USA) was added to each well of the plate, followed by 12 h incubation at 37°C. IgG N-glycans were enriched and purified from the enzyme solution by using a PGC-containing 96-well plate as described previously (1). In brief, the PGC-containing 96-well plate was first conditioned with 200 μL (for each well) of 0.1% (v/v) trifluoroacetic acid (TFA) in 80% acetonitrile (ACN)/H2O (v/v), followed by 0.1% TFA (v/v) in H_2O. The enzyme-released glycans solution was subsequently loaded to the PGC-containing 96-well plate three times to allow complete N-glycans binding. Then the plate was washed with H_2O to remove the salts and other contaminants. The purified N-glycans derived from IgG were finally eluted into the collection plate using 0.05% TFA(v/v) in 25% ACN/H2O (v/v).

MALDI-TOF MS Analysis

Before sample detection, a calibration standard of peptides (Calibration Standard II, Bruker Daltonics, Bremen, Germany) was used for external calibration. Next, the IgG N-glycans were analyzed by MALDI-TOF MS as previously described with minor modification (1, 3). Briefly, 1 μL of enriched, purified glycans sample was spotted onto an MTP BigAnchor 384BC MALDI target plate (Bruker Daltonics). Then 1 μL of matrix consisting of 5 mg/mL super-DHB in 50% ACN/H2O was mixed with the sample on the plate and allowed to dry by air, followed by recrystallization with 0.2 μL of pure ethanol. All mass spectrometry analyses were performed on a rapifleX MALDI-TOF mass spectrometer with a Smartbeam 3D laser in positive ion reflection positive (RP) mode, controlled by flexControl 4.0 (Bruker Daltonics). Sample measurements were performed with a mass range setting from *m/z* 1000 to m/z 3500. For each spectrum, 3000 laser shots were accumulated using a complete sample random walk with 200 shots per raster spot at a laser frequency of 5000 Hz.

Data Processing and Statistical Analysis

The pre-processing of MALDI-TOF MS data was performed according to our previous paper (2, 3). Briefly, MALDI-TOF MS spectra of the two cohorts were smoothed, baseline corrected, and transformed to .XY format by using flexAnalysis software (Bruker Daltonics). Using the in-house developed software MassyTools (version 0.1.8.1.2) (4), transformed .XY profiles of the cohorts were re-calibrated. Next, we used the Glyco-Peakfinder tool of GlycoWorkbench to generate putative glycan compositions, and the putative glycan compositions were validated again by previous literature (2, 5, 6). Briefly, peaks (isotope clusters) with a signal-to-noise ratio (S/N) above nine and good isotopic patterns were listed. Next, these peaks (analytes) were annotated with glycan compositions in GlycoWorkbench (version 2.1 stable build 146) using the Glyco-Peakfinder tool (5, 6). The generated glycan compositions were then confirmed/validated by previous literature (2), which applied the same protocols for IgG glycan analysis as in the present study. Of note, if multiple cation adduct signals such as $[M + Na]^+$ and $[M + K]^+$ were present simultaneously, all these

related adducts should be included in the quantification (2). The intensities for the putative N-glycan structures were finally extracted as background-corrected area for each spectrum of the samples using MassyTools. Derived glycan traits including Gal-ratio (representing the level of IgG galactosylation), fucosylation (F), agalactosylation (agal), and bisecting type neutral N-glycans (BN) were calculated based on the same structural features. The formulas used for the calculation: Agalactosylation=H3N4F1+H3N5F1;Fucosylation=H3N4F1+H4N4F1+H3N5F1+H5N4F1+H4N5F1+H5N5F1; Bisecting type neutral N-glycans=H3N5F1+H4N5F1+H5N5F1; Gal-ratio=H3N4F1/(H4N4F1+2*H5N4F1) (H = hexose; N = N-acetylhexosamine; F = deoxyhexose (fucose)). A glyco-panel was constructed by combining altered single N-glycans between cases and controls through logistic regression using SPSS (version 19.0). In this process, the group was set as the dependent variable, and the altered glycan traits were set as concomitant variables. Prediction probability generated during the logistical regression process was defined as glyco-panel. The following statistical analyses were performed with GraphPad Prism 7 and SPSS. Data quality was assessed and controlled by the inclusion of a standard plasma sample and calculating the average value, standard deviation (SD), and the relative SD (CV) for all glycans detected. The diagnostic potential of the IgG N-glycans detected in the present study was assessed by first performing statistical tests. Differences between two biological groups were assessed using the Mann-Whitney U test (non-normally distributed data), and the significance threshold of p-values is 0.004 (= p-value of 0.05 after multiple testing correction for 12 directly glycan traits). Differences between the three groups were evaluated by one-way analysis of variance (ANOVA) with Bonferroni correction for multiple comparisons using GraphPad Prism 7. Glycan traits resulting in statistically significant p-values were further evaluated by receiver-operator-characteristics (ROC) test to assess the specificity, sensitivity, and accuracy of the candidate IgG N-glycans using GraphPad Prism 7. The significance of the generated values of area-under-the-curve (AUC) was assigned. If the AUC values were greater than 0.9, the tests were considered “highly accurate,” while values between 0.8 and 0.9 were deemed “accurate.” When the AUC values were between 0.7 and 0.8, the tests were concluded to be “moderately accurate”. An “uninformative” test resulted in an AUC value that was between 0.5 and 0.7 (2, 7).

References

1. Ren S, Zhang Z, Xu C, Guo L, Lu R, Sun Y, et al. Distribution of IgG galactosylation as a promising biomarker for cancer screening in multiple cancer types. *Cell Res* (2016) 26(8):963-6. Epub 2016/07/02. doi: 10.1038/cr.2016.83.

2. Qian Y, Wang Y, Zhang X, Zhou L, Zhang Z, Xu J, et al. Quantitative analysis of serum IgG galactosylation assists differential diagnosis of ovarian cancer. *J Proteome Res* (2013) 12(9):4046-55. Epub 2013/07/17. doi: 10.1021/pr4003992.

3. Zhang Z, Westhrin M, Bondt A, Wuhrer M, Standal T, Holst S. Serum protein N-glycosylation changes in multiple myeloma. *Biochim Biophys Acta Gen Subj* (2019) 1863(5):960-70. Epub 2019/03/08. doi: 10.1016/j.bbagen.2019.03.001.

4. Jansen BC, Reiding KR, Bondt A, Hipgrave Ederveen AL, Palmblad M, Falck D, et al. MassyTools: A High-Throughput Targeted Data Processing Tool for Relative Quantitation and Quality Control Developed for Glycomic and Glycoproteomic MALDI-MS. *J Proteome Res* (2015) 14(12):5088-98. Epub 2015/11/14. doi: 10.1021/acs.jproteome.5b00658.

5. Maass K, Ranzinger R, Geyer H, von der Lieth CW, Geyer R. "Glyco-peakfinder"--de novo composition analysis of glycoconjugates. *Proteomics* (2007) 7(24):4435-44. Epub 2007/12/12. doi: 10.1002/pmic.200700253.

6. Ceroni A, Maass K, Geyer H, Geyer R, Dell A, Haslam SM. GlycoWorkbench: a tool for the computer-assisted annotation of mass spectra of glycans. *J Proteome Res* (2008) 7(4):1650-9. Epub 2008/03/04. doi: 10.1021/pr7008252.

7. Swets JA. Measuring the accuracy of diagnostic systems. *Science* (1988) 240(4857):1285-93. Epub 1988/06/03. doi: 10.1126/science.3287615.

Supplemental Figures

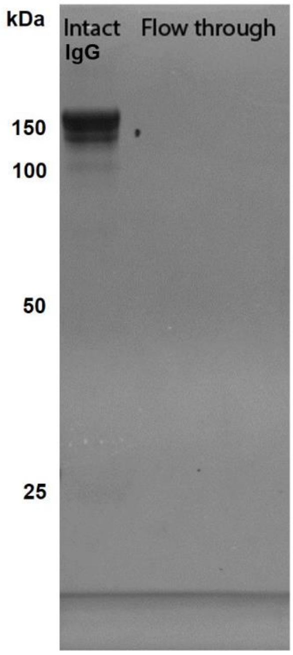

Figure S1 SDS-PAGE gel confirming the IgG purity after protein A enrichment. IgG is captured from plasma, and the flowthrough and eluate are collected.

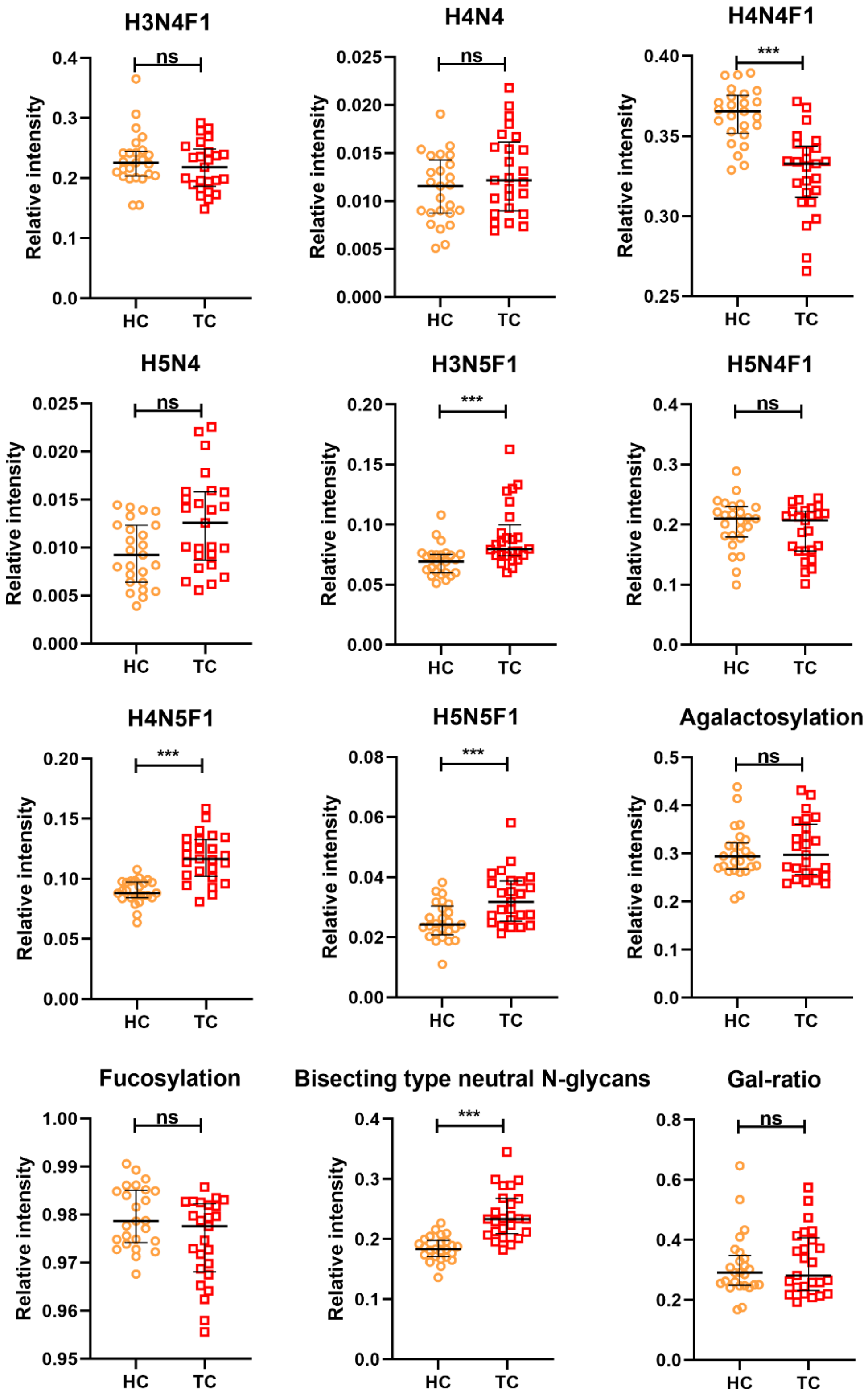

Figure S2 The scatter plots of all directly detected and derived glycan traits in thyroid cancer (TC) and healthy controls (HC) for the discovery cohort. The whiskers represent “median with IQR”. *** = p-value < 0.001, ** = p-value < 0.01, * p-value < 0.05, ns = not significant (after Bonferroni correction).

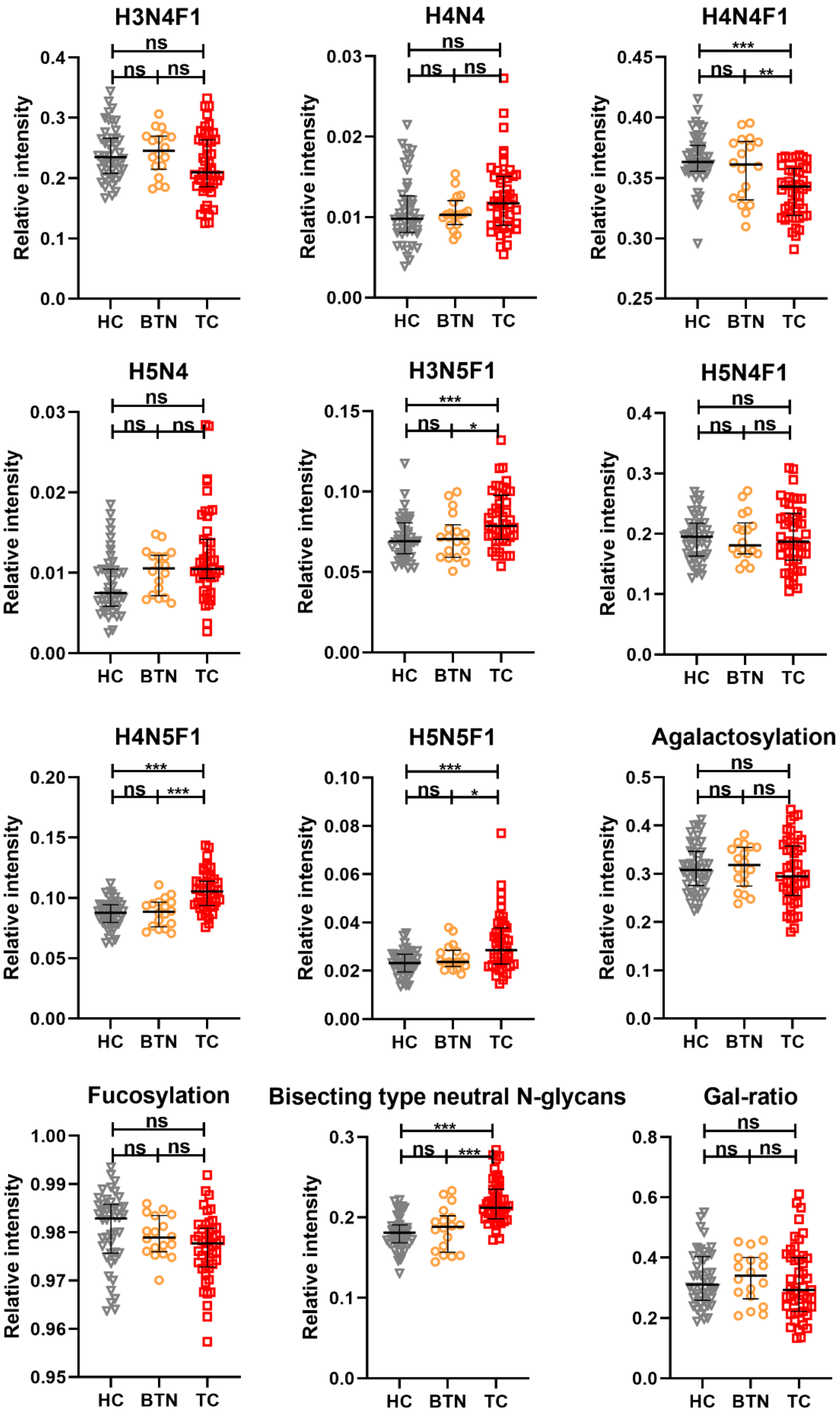

Figure S3 The scatter plots for all directly detected and derived glycan traits in thyroid cancer (TC), benign thyroid nodules (BTN) and healthy controls (HC) for the validation cohort. The whiskers represent “median with IQR”. *** = p-value < 0.001, ** = p-value < 0.01, * p-value < 0.05, ns = not significant (after Bonferroni correction).

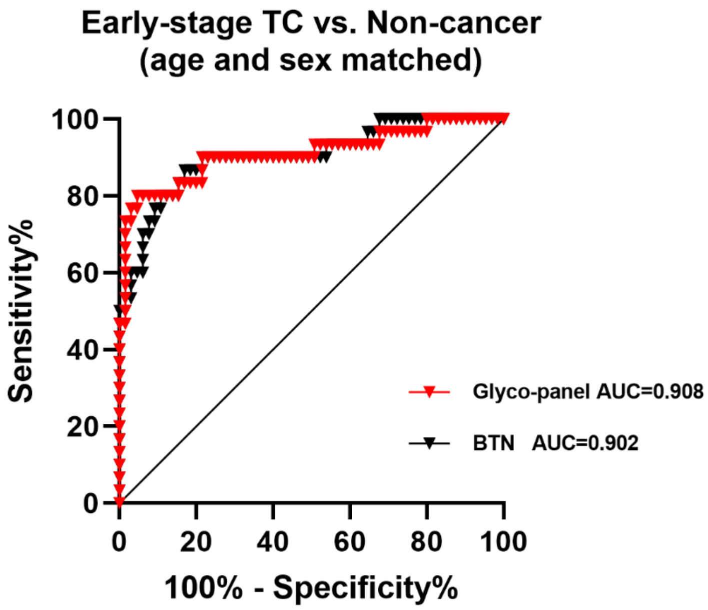

Figure S4 ROC analysis for age- and sex- matched early-stage thyroid cancer (TC) and non-cancer controls.
